# Association of residential dampness and mold with respiratory tract infections and bronchitis: a meta-analysis

**DOI:** 10.1186/1476-069X-9-72

**Published:** 2010-11-15

**Authors:** William J Fisk, Ekaterina A Eliseeva, Mark J Mendell

**Affiliations:** 1 Indoor Environment Department, Environmental Energy Technologies Division, Lawrence Berkeley National Laboratory, 1 Cyclotron Road 90R3058, Berkeley, CA, USA

## Abstract

**Background:**

Dampness and mold have been shown in qualitative reviews to be associated with a variety of adverse respiratory health effects, including respiratory tract infections. Several published meta-analyses have provided quantitative summaries for some of these associations, but not for respiratory infections. Demonstrating a causal relationship between dampness-related agents, which are preventable exposures, and respiratory tract infections would suggest important new public health strategies. We report the results of quantitative meta-analyses of published studies that examined the association of dampness or mold in homes with respiratory infections and bronchitis.

**Methods:**

For primary studies meeting eligibility criteria, we transformed reported odds ratios (ORs) and confidence intervals (CIs) to the log scale. Both fixed and random effects models were applied to the log ORs and their variances. Most studies contained multiple estimated ORs. Models accounted for the correlation between multiple results within the studies analyzed. One set of analyses was performed with all eligible studies, and another set restricted to studies that controlled for age, gender, smoking, and socioeconomic status. Subgroups of studies were assessed to explore heterogeneity. Funnel plots were used to assess publication bias.

**Results:**

The resulting summary estimates of ORs from random effects models based on all studies ranged from 1.38 to 1.50, with 95% CIs excluding the null in all cases. Use of different analysis models and restricting analyses based on control of multiple confounding variables changed findings only slightly. ORs (95% CIs) from random effects models using studies adjusting for major confounding variables were, for bronchitis, 1.45 (1.32-1.59); for respiratory infections, 1.44 (1.31-1.59); for respiratory infections excluding nonspecific upper respiratory infections, 1.50 (1.32-1.70), and for respiratory infections in children or infants, 1.48 (1.33-1.65). Little effect of publication bias was evident. Estimated attributable risk proportions ranged from 8% to 20%.

**Conclusions:**

Residential dampness and mold are associated with substantial and statistically significant increases in both respiratory infections and bronchitis. If these associations were confirmed as causal, effective control of dampness and mold in buildings would prevent a substantial proportion of respiratory infections.

## Background

Dampness and mold in buildings have been associated in many studies with adverse respiratory health effects. A number of qualitative summaries of this literature are available [1-3]. In their review, the Institute of Medicine (IOM) of the National Academy of Sciences found sufficient evidence to document an association between qualitatively assessed indoor dampness or mold and upper respiratory tract symptoms, cough, wheeze, and asthma symptoms in sensitized persons [2]. A later review by the World Health Organization (WHO), including additional studies, expanded the documented associations to include asthma development, current asthma, dyspnea, and respiratory infections. While both reviews concluded that excessive indoor dampness was an important public health problem meriting prevention and remediation, neither review produced quantitative summaries of association between dampness or mold and specific health outcomes.

Two prior quantitative meta-analyses have been published on indoor dampness and mold and selected health effects. In 2007, Fisk et al. [4] quantitatively summarized the associations of home dampness and mold with a set of respiratory and asthma-related health effects, based on available studies published in peer-reviewed journals in English [4]. Health outcomes included were upper respiratory tract symptoms, cough, wheeze, asthma diagnosis ever, current asthma, and asthma development. The meta-analyses produced central estimates of ORs ranging from 1.34 to 1.75 for these health outcomes, with 95% confidence intervals (CIs) excluding the null in nine of ten instances. Antova et al. [5] analyzed pooled data from 12 European cross-sectional studies of visible mold in residences and respiratory or allergic health outcomes of children. Outcomes included bronchitis, wheeze, asthma, nocturnal dry cough, morning cough, sensitivity to inhaled allergens, hay fever, and "woken by wheeze." Central estimates of ORs ranged from 1.30 to 1.50, with all 95% CIs excluding the null.

Thus while prior non-quantitative reviews have reported consistent associations between dampness or mold and respiratory infections, no quantitative meta-analysis of this relationship has been reported. A substantial number of epidemiologic studies on dampness or mold and respiratory infections are available for this purpose.

Respiratory (tract) infections are generally considered to include infections of the lower and upper respiratory tract, and otitis media. Lower respiratory tract infections include pneumonia, acute bronchitis, and acute exacerbation of chronic bronchitis. While acute bronchitis is generally caused by an infection, chronic bronchitis is generally non-infectious in origin. Upper respiratory tract infections are acute infections of the nose, sinuses, and throat. Otitis media, an infection or inflammation of the middle ear often resulting from a prior upper respiratory tract infection, can be bacterial or viral in origin.

The burden of morbidity and mortality and the financial costs of respiratory tract infections are enormous. Little effective prevention is currently possible outside of two strategies: attempting to avoid contact with or spreading of infectious agents in aerosols, droplets, or surfaces, such as by hand washing, avoiding infected individuals, avoiding face-touching, and covering sneezes; and vaccination for influenza and pneumococcal pneumonia. It is important to determine whether avoidance of dampness and mold can provide another means of reducing respiratory tract infection. As a step toward that goal, we performed a quantitative meta-analysis to summarize findings in the peer-reviewed medical literature on associations between dampness or mold in residences and respiratory tract infections or bronchitis.

## Methods

Our search for published articles involved several strategies (see details in Additional File 1, Appendix 1): an online search of PubMed, an online search of the journal Indoor Air, and a manual search of the reference list in the publication "World Health Organization Guidelines on Dampness, Mold, and Health" [3].

Papers meeting all of the following criteria were selected for use in the meta-analyses:

1) published in a refereed archival journal in English.

2) based on original data; i.e., not a review article or meta-analysis.

3) reported effect estimates as odds ratios (ORs) or risk ratios (RRs), with confidence intervals (CIs) estimated from statistical analyses.

4) included as risk factors qualitatively assessed dampness, mold, or dampness and mold in residences, whether in detached homes or multifamily housing (dormitory rooms were accepted as homes)

5) included one or more of the health outcomes listed in Table 1 (see below).

**Table 1:** Health outcomes from reviewed studies, grouped into outcome categories used in meta-analyses

Category in Meta-Analysis	Number of studies	Outcomes from Individual Studies Included in Each Category
Bronchitis (all: acute or chronic)	13	bronchitis, bronchitis in the prior year, current bronchitis, obstructive bronchitis, chronic bronchitis; doctor diagnosed bronchitis in the past year; bronchitis indicated by cough and phlegm ≥ 3 months for at least two consecutive years, bronchitis times per year

Respiratory infection group	19	airway infection last month; sinus or ear infection with antibiotic use; cold; common cold; > 4 (or > 6) colds in last 12 months; frequent childhood respiratory infections; sinusitis; tonsillitis; acute upper respiratory tract infection in past 12 months; tonsillopharyngitis, croup, bronchitis, or bronchiolitis diagnosed by doctor; chest cold; consulting general practitioner for acute respiratory tract infection (with wheeze); sum of episodes of tonsillitis, sinusitis, otitis, bronchitis; one or more episodes of bronchitis or pneumonia; tonsillitis, otitis media, sinusitis, bronchitis, or pneumonia at least once; chest cold with wheeze; otitis media; pneumonia; bronchitis times per year

Respiratory infections excluding otitis media	17	same as listed in cell above excluding otitis media

Respiratory infection group excluding nonspecific upper respiratory infection	15	sinus or ear infection with antibiotic use; sinusitis; tonsillitis; tonsillopharyngitis, croup, bronchitis, or bronchiolitis diagnosed by doctor; sum of episodes of tonsillitis, sinusitis, otitis, bronchitis; one or more episodes of bronchitis or pneumonia; tonsillitis, otitis media, sinusitis, bronchitis, or pneumonia at least once; otitis media; pneumonia; bronchitis times per year

6) included at least ten buildings, if building-level exposures were used.

We performed one set of analyses including only results from studies that controlled for potential confounding by the following factors via study design or analysis method: age, gender, smoking (e.g., active smoking, smoking in home, smoking by mother during pregnancy), and some measure of socioeconomic status (SES). We considered studies with populations limited to home owners, university students, or university employees as adequately controlled for SES. We also considered the reporting of no significant association between an outcome and a potential confounder as equivalent to controlling for that confounder. In another set of unrestricted analyses, we did not require control for these potential sources of confounding, although most of the added studies controlled for all but one of these factors.

For papers which reported strength of association as RRs instead of ORs, we included RRs as if they were ORs for the primary analysis. RRs approximate ORs well when outcome prevalence is low; however, we also performed an analysis excluding RR values.

Ideally, a meta-analysis would utilize input data only from studies with the same precisely defined risk factor, health outcome, and population. As this was not possible, we used input data from studies that were as similar as practicable, all in residences. The following risk factors were accepted: dampness, water damage, visible mold, mold odor, or flooding - all in the whole home, main living area, or bedroom. We did not distinguish among dampness, mold, dampness or mold, and dampness and mold as risk factors. Our rationale - visible mold is always considered the result of excess dampness whether or not the dampness is reported, and excess dampness is very often accompanied by mold, although the mold may not be visible. Thus, it was not possible to make a clear distinction among these risk factors. Excluded as inputs were ORs for condensation (a less certain indication of potential microbial contamination), ORs per unit area of visible mold or water damage, ORs for "suspected moisture problem," and ORs for higher measured airborne concentrations of molds, bacteria, ergosterol, glucan, or endotoxin. The included studies had either adults or children as subjects. Presence of dampness and/or mold was determined in each study by either the occupants or the researchers. We did not distinguish between occupant-reported dampness and/or mold and researcher-reported dampness and/or mold.

The categories of health outcomes constructed for meta-analyses were respiratory infection group, respiratory infection group excluding otitis media, and bronchitis (acute, chronic, or not clearly characterized as acute or chronic). The respiratory infection group outcomes involved viral or bacterial infections; we excluded from consideration respiratory infections by fungi which occur primarily in people with compromised immune systems. The respiratory infection and bronchitis outcome categories overlap, with some studies of respiratory infections including bronchitis or episodes of bronchitis within their definition of a respiratory infection. We included separate bronchitis outcomes in the respiratory infection group only if the definition stated or suggested acute bronchitis. The category of bronchitis includes acute bronchitis, normally the result of an acute respiratory infection, and chronic bronchitis, which may be unrelated to an infection. Most papers did not provide sufficient information to allow classification of the bronchitis as acute or chronic.

For respiratory infections, we also produced summary estimates separately for studies of children and of adults (omitting the one study that included both). In addition, we produced a summary estimate for the respiratory infections group after excluding findings for a set of relatively nonspecific upper respiratory outcomes that seemed most susceptible to inclusion of allergic or irritant symptoms. This excluded findings such as for common cold, chest cold with wheeze, acute upper respiratory infections, acute respiratory tract infections, respiratory infections, and airway infections. We did not exclude throat infections, sinusitis, tonsillitis, otitis, or the various lower respiratory infections.

We applied random effects models [6] to derive central estimates and confidence limits for associations of the health outcomes with dampness and mold as reported by the multiple published studies which varied in symptom definitions, subjects, and locations. The approach used was the same as in a prior meta-analysis of dampness and mold with respiratory and asthma outcomes [4]. In each meta-analysis model, we included multiple ORs from single studies that reported more than one OR for different but correlated risk factors (e.g., visible mold; dampness), different health outcomes (e.g., respiratory infection, common cold), or both; e.g., ORs in one study for visible mold with bronchitis, dampness with bronchitis, visible mold with respiratory infections, and dampness with respiratory infections. Random effects models adjusting for possible within-study correlations were used in our primary analyses. In addition, we used the procedure PROC MIXED in SAS (ver 9.2, SAS Institute Inc., Cary, NC), which allows fixing the within-study variances (matrix R in SAS) while estimating between-study variance (matrix G in SAS).

ORs and 95% CIs reported in each reviewed study were first transformed to the log scale. The transformed results for each outcome category were then combined using a random effects model. The model accounting for the correlation between multiple results within studies was

(1) $$y_{ij}\sim N(\beta_{0}+\beta_{0i},\sigma_{ij}^{2})$$

where:

*y_ij _*is the *ln *OR in the *j*th sub-study of the *i*th study;

*β*_*0 *_is the fixed effect across all studies;

*β*_*0i *_is the random effect in the *i*th study. *β*_*0i *_~ *N*(0, σ*^2^), where:

σ*^2 ^is the between-study variance; and

$\sigma_{ij}^{2}$ is the within-study variance, calculated from the log CI.

Results based on the model described above were compared to those obtained from secondary analyses using fixed effects models that assumed independence of multiple ORs within individual studies. Additional models were constructed that omitted the reported RR values. For final models, we assessed heterogeneity of study-specific effect estimates using the meta command in STATA to estimate the Q statistic and associated p-value. Where the p-value for heterogeneity was <0.05 for both the full and restricted sets of findings, we further explored possible sources of heterogeneity by conducting sensitivity tests, and performing tests of heterogeneity for various subsets of findings, as feasible.

Funnel plots were produced to check for evidence of publication bias. If the plot for an outcome showed asymmetry only among less precise (generally smaller) studies, suggesting that smaller studies without positive findings were less likely to have been published, then an alternate analysis was performed. This excluded the set of smaller studies exhibiting asymmetry, in order to produce presumably less biased summary estimates based only on the more completely reported, more precise studies.

## Results

Overall, 23 studies were selected for inclusion in these meta-analyses. Table 1 provides the number of studies for each health outcome category and the specific outcomes from reviewed studies included in each category. Table 2 identifies the studies in each health outcome category. It was not possible to summarize findings for acute bronchitis separately, as too few studies reported findings for an outcome clearly restricted to acute or infectious bronchitis.

**Table 2:** Studies included in the meta-analyses

Study		Health **Outcomes**^**#**^	Study Type	Number of Subjects^	Controlled **for Age, Sex**, Smoking and Socioeconomic Status
Bakke et al. 2007	[29]	RI	cross sectional	173	Yes

Biagini et al. 2006	[10]	RI	birth cohort	585	Yes

Brunekreef et al. 1989	[30]	B	cross sectional	4,625	Yes

Dales et al. 1991	[31]	B	cross sectional	13,495	No

Diez et al. 2003	[32]	B	birth cohort	172 - 178	No

du Prel et al. 2006	[33]	RI, B	cross sectional	5,757 - 20,059	Yes

Ekici et al. 2008	[34]	RI, B	cross sectional	9,610 - 9,853	Yes (BR) No (RI)

Haverinen et al. 2001	[8]	RI, B	cross sectional	1,017	Yes

Karevold et al. 2006)	[35]	RI	cross sectional	275 - 737	Yes

Kilpelainen et al. 2001	[36]	RI	cross sectional	9,765 - 10,504	No

Koskinen et al. 1999	[37]	RI, B	cross sectional	57 - 147	No

Li and Hsu 1996	[38]	RI, B	cross sectional	1,340	Yes

Pettigrew et al. 2004	[39]	RI*	birth cohort	806	No

Pirhonen et al. 1996	[40]	RI, B	cross sectional	1,460	Yes

Rylander and Megevand 2000	[41]	RI, B**	cross sectional	304	No

Spengler et al. 1994	[42]	B	cross sectional	12,842	No

Spengler et al. 2004	[43]	RI, B	cross sectional	5,951	Yes

Stark et al. 2003	[7]	RI	birth cohort	499	Yes

Strachan 1988	[44]	RI	cross sectional	873	No

Sun et al. 2009	[45]	RI	cross sectional	3,436	Yes

van Gageldonk-Lafeber et al. 2007	[9]	RI	case-control	626	No

Yang et al. 1997	[46]	RI, B	cross sectional	4,164	Yes

Yang et al. 1999	[47]	RI*	case control	438	Yes

^# ^RI = respiratory infection group, B = bronchitis (acute, chronic, or uncharacterized as acute or chronic), * Outcome is otitis media, most often accompanied by an upper respiratory infection

^ used for inputs to meta-analyses ** Bronchitis times per year assumed to be acute/infectious bronchitis

Major results from the meta-analyses of all eligible studies, regardless of control for confounding, are provided in column 2 of Table 3. For the two primary outcomes, bronchitis and respiratory infections, central estimates of ORs were 1.44 and 1.45. For these and all other subcategories in Table 3, 95% CIs excluded the null. P-values for heterogeneity for both were <0.0001. For bronchitis and the respiratory infection group, central estimates changed little (by less than 0.01) when the models were restricted to studies that controlled for age, gender, smoking, and socioeconomic status (column 3); however, with this restriction the p-value for heterogeneity for bronchitis increased to 0.12. Estimates (not shown) derived from fixed effects models were also very similar to the estimates in Table 1 - the maximum change in central estimate OR was 0.04. For the respiratory infection group, excluding RR values reported by two studies [7,8] changed the central estimate by less than 0.01 and confidence interval endpoints by 0.03 or less.

**Table 3:** Key results of the meta-analyses, with results of tests for heterogeneity

Health Outcome	All Studies		Studies Controlling for All Four Key Confounders		
	**Summary Estimate OR (95% CI)**	**p-Value Hetero-geneity**	**Summary Estimate OR (95% CI)**	**p-Value Hetero-geneity**	**Attributable Risk Proportion^#^**

Bronchitis	1.45 (1.34 - 1.56)	< 0.0001	1.45 (1.32 - 1.59)	0.12	8.3 - 18.4%

Respiratory infection group	1.44 (1.32 - 1.58)	< 0.0001	1.44 (1.31 - 1.59)	< 0.0001	

Respiratory infections excluding otitis media	1.43 (1.31 - 1.56)	< 0.0001	1.40 (1.29-1.52)	< 0.0001	

Respiratory infections excluding common cold and nonspecific upper respiratory infections	1.42 (1.26 - 1.60)	0.01	1.50 (1.32 - 1.70)	0.07	9.1 - 20%

Common cold or acute upper respiratory infection	1.38 (1.21 - 1.57)	0.009	1.38 (1.13 - 1.67)	0.002	

Respiratory infections (children or infants)	1.48 (1.34 - 1.62)	0.16	1.48 (1.33 - 1.65)	0.09	8.8 - 19.4%

Respiratory infections (adults)	1.50 (1.22 - 1.83)	< 0.0001	1.49 (1.14 - 1.95)	< 0.0001	

^# ^estimated for findings restricted to studies controlling for four key confounders and assuming a range of 20-50% of houses with dampness or mold; provided only for estimates with p-value for heterogeneity >0.05.

A series of models excluding each finding sequentially did not identify highly influential single findings. The two most extreme findings (ORs of 0.48 [9] and 5.1 [10]) were not from large studies, and did not have major influence. Additional models were constructed with specific subgroups of respiratory infection outcomes (Table 3). For these subgroups, when restricted to studies with control of at least the four key confounding factors, modeling outcomes of respiratory infections excluding otitis media did not much change the estimate or decrease heterogeneity. Modeling outcomes of respiratory infections excluding common cold and nonspecific upper respiratory infections increased the central estimate to 1.50 and decreased heterogeneity (p = 0.07). Restricting the model to only common cold or acute upper respiratory infection (excluding several findings of unspecified respiratory infections), the central estimate was 1.38, but with high heterogeneity. Constructing separate overall respiratory infection group models for children/infants and for adults led to similar ORs of 1.48 and 1.49, with decreased heterogeneity (p = 0.09) only for children/infants. Other study factors potentially contributing to heterogeneity included statistical adjustment for subject atopy, parental atopy, or presence of furry pets, and whether assessment of environmental dampness was conducted by researchers or participants. Numbers in these subgroups were small, and inspection of estimates revealed no clear potential to influence heterogeneity.

**Figure 1:**
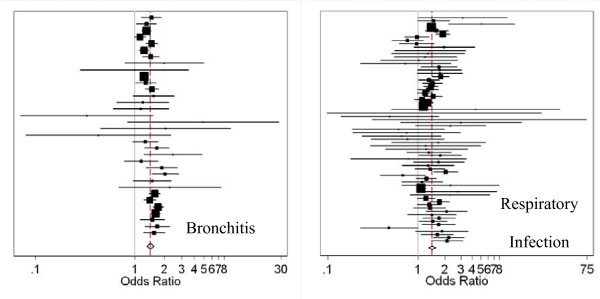
**Odds ratios and confidence intervals from all studies meeting the less restricted eligibility criteria and from a meta-analysis of these studies performed using the random effects model and assuming dependent estimates within studies**. The width of the boxes (some so small they appear as points) is proportional to the precision of the study and the ends of the horizontal lines represent lower and upper 95% confidence limits. The left vertical line marks an odds ratio of 1.0, corresponding to no increased risk, while most of the reported odds ratios are greater than unity indicating an increase in risk with dampness and mold. The central estimate from the meta-analysis is indicated by the right vertical line and the left- and right-side points of the diamond at the bottom of the figure indicate the lower and upper 95% confidence limits from the meta-analyses.

Figure 1 shows forest plots with adjusted ORs and 95% CIs for the associations of respiratory infections and bronchitis with dampness and mold as reported in the original studies. Figure 1 also shows the summary estimates produced in the meta-analyses using random effects models with all studies listed in Table 2.

**Figure 2:**
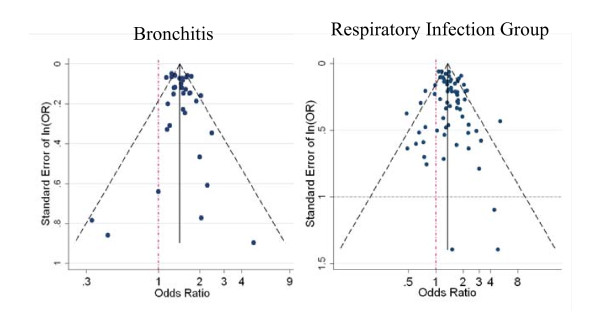
**Funnel plots for bronchitis and the respiratory infection group**. The horizontal line in the plot for the Respiratory Infection Group indicates the line (Standard Error = 1) below which asymmetric data points were omitted in a secondary analysis.

Funnel plots for the respiratory infection group and bronchitis are shown in Figure 2. No asymmetry was evident for bronchitis. The asymmetry in data points for the respiratory infection group, i.e., the absence of published ORs less than 1.0 produced by less precise (generally smaller) studies, suggested possible publication bias. When we excluded study results with standard errors greater than 1.0 (the set with asymmetric estimates), the revised estimate for this outcome differed by only 0.01 from the estimate in Table 1, suggesting that publication bias had little effect on the central estimates.

## Discussion

The results of these meta-analyses indicate that building dampness and mold are associated with moderate but statistically significant increases in respiratory infections and bronchitis. The central estimates and confidence limits for these associations were stable across different modeling strategies: adding studies that controlled for fewer confounding variables, assuming independence of multiple estimates from the same studies, and omitting included RRs. Also, analyses suggest that publication bias likely had little impact on these estimates.

The statistical associations reported here do not document that dampness and mold are causally related to the bronchitis and respiratory infections. Building dampness itself is unlikely to directly cause adverse health effects. If these associations are confirmed as causal, exposure to one or more dampness-related agents, either microbiologic or chemical, is likely to be ultimately implicated. However, the consistent evidence of adverse health effects from a substantial number of studies that have controlled for key potential confounders, along with the moderately strong associations and the limited evidence of publication bias, provide initial evidence for causal links between these health effects and some dampness related agent(s).

Evidence for relationships of dampness or mold with respiratory infections and bronchitis has strengthened -- initially anecdotal, now documented in multiple observational studies. Within the past decade, there have been at least three major qualitative reviews of the associations of dampness and mold with health outcomes. An interdisciplinary Nordic review panel in 2001 [11] concluded "There also seems to be an association between dampness and.... airway infections." but this review provided no conclusions pertaining to the association of dampness with bronchitis. The IOM review in 2004 [2] made no conclusions relative to the association of dampness or mold with respiratory infections or bronchitis, but stated "Healthy persons exposed to dampness or moldy indoor environments sometimes report that they are more prone to respiratory infections...." The most recent review, by WHO in 2009 [3], concluded that there is sufficient evidence to document an association of dampness and dampness-related agents with respiratory infections, but only limited or suggestive evidence of an association for bronchitis. The results of the present quantitative meta-analyses are consistent with the WHO findings for respiratory infections, but imply more strongly that dampness and mold are associated with bronchitis.

Prior quantitative meta-analyses on health effects of dampness and mold have not included a category for respiratory tract infections overall. The meta-analysis by Antova et al. [5] on visible mold in residences and bronchitis in children, based on a set of similar European studies, reported an OR (95% CI) of 1.38 (1.29-1.47). This compares well to the summary OR reported here for dampness or mold in residences and bronchitis, based on the larger medical literature, of 1.45 (1.32-1.59).

The outcome categories included in this review contain a variety of specific diseases, with all but chronic bronchitis caused by a range of infectious organisms. We will consider biologic plausibility of the associations reviewed here separately for the infectious and non-infectious mechanisms.

Respiratory infections include upper and lower respiratory tract infections and otitis media. Upper respiratory tract infections include common colds, pharyngitis (sore throat), and sinusitis. Most are caused by viruses such as rhinovirus, coronavirus, adenovirus, or respiratory syncytial virus, although a minority of cases is caused by bacteria [12]. Otitis media, an infection or inflammation of the middle ear often resulting from a prior upper respiratory tract infection, can be bacterial or viral in origin [13].

Lower respiratory tract infections, including pneumonia, acute bronchitis, and acute exacerbation of chronic bronchitis, can result from a variety of causal organisms, including *Haemophilus influenza*, *Streptococcus pneumoniae*, and *Moraxella catarhalis *[14]. Pneumonia is an inflammation of the lung, caused usually by an infection from bacteria, virus, or fungi, but sometimes by accidental inhalation of other substances [15]. Bronchitis, an inflammation of the mucus membranes of the bronchi, can be acute or chronic. Acute bronchitis often occurs in conjunction with viral infections such as common cold (e.g., rhinovirus, adenovirus), respiratory syncytial virus, or influenza, with a minority of cases caused by bacterial infections. In contrast, chronic bronchitis is generally caused not by respiratory infection, but by recurring injury or irritation to the lining of the bronchi, such as from tobacco smoke or irritating dust or fumes [16].

An evident increase in respiratory infections in association with dampness or mold could occur from increased numbers of infections, or from more serious infections that are more clinically apparent; either might result from impairment of immune defenses. Although the specific exposures occurring in the reviewed studies are not known, and although it has not been demonstrated that exposures to microbial toxins in typical damp or moldy houses can suppress immune response in humans, potential underlying mechanisms can be suggested. Studies both in vitro and in vivo have demonstrated inflammatory and immunosuppressive responses to the spores, metabolites, and components of specific microorganisms found in damp buildings [2,3]. Repeated activation of immune responses and inflammation from microbiologic exposures may contribute to inflammation-related diseases, and the resulting inflamed mucosal tissue may provide a diminished barrier to respiratory infections. Observed synergistic interactions in toxicologic studies among microbial agents present in damp buildings, including specific fungi, actinomycetes, and amoebae (e.g. [17,18]) suggest that immunotoxic effects of fungal and bacterial strains typically found in damp buildings may be potentiated during joint exposures. This could explain lack of evident associations for specific exposures. Thus, some biologic plausibility is evident even in the absence of consistent associations between human exposures to specific microorganisms or microbial components or products and respiratory infections in healthy individuals.

For chronic bronchitis, more often caused by chronic exposures to irritants and inflammatory agents, the immunostimulatory and inflammatory agents and allergens in some molds and other dampness-related microbial agents may explain or contribute to the associations [2,3]. Also, dampness in building materials can increase the emission rates and indoor concentrations of some chemicals [2], such as formaldehyde, which could cause irritation or inflammation [19,20].

Our analysis is subject to multiple limitations. Publication bias in the selection of available studies remains a possibility despite the limited evidence of publication bias effects described above. Estimates from random effects models should be interpreted with caution when the number of observations is small, as in some sub-analyses reported here. The test of heterogeneity used here has low power to reject the null hypothesis when the number of included findings is small.

The respiratory infections category used in this analysis is broad, including outcome definitions of various types of lower respiratory infections that include acute bronchitis; common cold; mixes of lower and upper respiratory infections; and upper respiratory infections including otitis. There were not sufficient numbers of most outcomes for separate analyses. We have separately estimated summary measures of effect for bronchitis (acute or chronic), respiratory infections overall, and various subsets of respiratory infections. It is possible that some disease caused by allergy or irritation, especially in the upper respiratory tract, was classified erroneously as respiratory infection. Since allergy and irritation are known to be associated with damp indoor spaces, this could have resulted in erroneously linking dampness and mold with respiratory infections. To check this possibility, we estimated risks for a restricted set of respiratory infections: including lower respiratory infections plus specific upper respiratory infections of tonsillitis, pharyngitis, sinusitis, and otitis, but excluding common cold and less specific upper respiratory infections (e.g., acute upper airway infections, airway infection, and frequent childhood respiratory infections), with the highest potential of being allergic or irritant outcomes misclassified as infections. Because this restriction of the respiratory infection outcomes increased the summary OR slightly from 1.44 to 1.50 (and reduced heterogeneity of findings), this potential misclassification is not likely to explain the elevated risk of infections found here with dampness or mold. Regarding the summary OR of 1.38 for common cold and acute upper respiratory infections, it is not clear how much allergic and irritant effects have been included with true upper respiratory infections. We did not estimate effects for a category of lower respiratory infections because these findings were mostly for acute bronchitis. There were only seven findings for pneumonia from three studies (ORs 0.79, 1.30, 1.33, 1.71, 1.77, 1.85, and 2.3), too few to allow confidence in a meta-analysis (estimated summary OR = 1.57), but suggestive of increased risk.

The substantial diversity of findings in the studies reviewed here was evident in the initial low p-values for heterogeneity. When acute bronchitis findings were restricted to studies adjusted for the four key confounding variables, the p-value for heterogeneity increased to 0.12. This suggests that heterogeneity for the unrestricted findings may have been due to scattered estimates from less well-adjusted studies. That the central OR estimate, 1.45, remained unchanged with this restriction suggests scatter in the unrestricted findings rather than systematic bias.

For the respiratory infection group, restriction to findings from more consistently adjusted models omitted many of the most extreme estimates (e.g., 0.48, 0.49, 4.4, 4.8), but did not decrease heterogeneity of the remaining findings. Exclusion of relatively nonspecific upper respiratory infections, which might be misdiagnosed allergic or irritant effects, increased the central estimate to 1.50 and decreased heterogeneity (p = 0.07), whereas the estimate for common cold or acute upper respiratory infection was 1.38. While substantial heterogeneity remained within many of the subgroups listed in Table 3, for those subgroups with little heterogeneity within, differences in OR were not large.

Because of the small number of available studies and the frequent use of outcomes containing multiple diseases, clear conclusions cannot be drawn about even associations with specific infectious diseases such as influenza. While the central estimate for common cold or acute upper respiratory infection of OR = 1.38, the lack of homogeneity in the included findings and the uncertain diagnosis makes this estimate only suggestive.

Most studies included here relied on occupant reporting of dampness and mold, a possible source of both systematic bias and error. However, two prior reviews have considered whether biased subjective response by building occupants in dampness studies might have positively biased the findings. The prior comparison by Fisk et al. of occupant-reported versus independent researcher-based assessments of dampness and mold in six studies [4] concluded that it is "very unlikely that the observed association of respiratory health effects with dampness and mold is a consequence of over-reporting of dampness and mold by occupants with respiratory symptoms." Bornehag et al. [11] reported that findings of studies with independent assessment of both dampness and health effects were similar to findings of studies with more subjective information sources.

The use of subjective, qualitative assessments of dampness and mold, even if not systematically biased, will misclassify actual causal exposures. However, these subjective metrics are currently the most useful correlates of health effects. Direct causal exposures related to dampness and mold have not yet been documented. Many quantified assessments of microbial exposures have been studied, and they have not shown consistent associations with specific health effects in healthy individuals [3]. This is likely because the specific causal exposures involved have either not yet been identified or not been well measured. Also, as Antova et al. say, visible molds "may better represent long-term exposure to moulds than direct measurements during a short sampling time [5]."

The majority of underlying data are from cross sectional studies that are subject to confounding and other limitations inherent in that study design, despite the attempts to control for known confounders. The resulting estimates are all less than 1.5, making their elevations especially susceptible to alternate explanation by unmeasured confounding factors and other biases rather than by dampness- or mold- related exposures. It is not clear what additional confounding variables might explain these findings consistently across studies. On the other hand, since the risk factors assessed in these studies are likely to be surrogates for unmeasured indoor dampness-related causal exposures, ORs for the true causal exposures would be higher.

The primary summary estimates reported here required that studies controlled at least for age, gender, smoking, and SES (although many included studies also controlled for other factors). If studies did not adequately control for all important confounders, biased estimates may have resulted. Evidence suggesting that substantial residual bias was unlikely comes from the paper by Antova et al. [5]. Only two of the 23 studies included here were among the 12 included in Antova et al. Yet findings for bronchitis here and in the pooled data analysis of over 58,000 children by Antova et al were very similar, even though Antova et al. adjusted for 13 potential confounding factors - age, gender, current smoker in household, maternal smoking during pregnancy, maternal and paternal education, household crowding, nationality, gas cooking, unvented gas/oil/kerosene heaters, birth order, "ever had a pet," and study area. Also, the analysis by Antova et al., when adjusted only for age, gender, and geographic area, gave similar estimates as when adjusted for many factors. Although the estimates included in Antova's summary for bronchitis had significant heterogeneity, estimates from all included studies exceeded 1.0, and CIs for nine of the 10 exceeded 1.0. Furthermore, Antova et al. performed a sensitivity analysis on potential heterogeneity on other variables such as season of questionnaire, age of subject, year of study, and response rate, and found little effect other than a significantly higher ORs for bronchitis in studies with above 80% response. Overall, this suggests that the relationships of bronchitis and various other respiratory outcomes to mold are not much confounded by the most obvious variables, and are not modified substantially by other key variables.

Respiratory tract infections, the most common infectious diseases in humans, have large health and cost consequences for individuals and for the public. Acute lower respiratory infections are the leading cause of death in children below five years old worldwide [14]. Community-acquired pneumonia (e.g., not hospital-acquired or in the immunosuppressed) is a major cause of hospitalization and morbidity and costs more than $17 billion dollars annually in the U.S. [15]. Otitis media is the most common bacterial infection in children, and is a major cause for antibiotic prescriptions [13]. Estimates of the prevalence of dampness or mold problems in houses are available from multiple sources, and include the following: at least 20% in European countries, the U.S., and Canada [2]; 14-40% in Europe, Russia, and North America [5]; and 50% in the U.S. [21].

Little effective prevention is currently possible for human respiratory infections outside of attempting to avoid contact with or spreading infections, vaccination for influenza and pneumococcal pneumonia, and possibly specific nutritional supplementation [22]. The few documented environmental risk factors for respiratory infections include environmental tobacco smoke [23], wood or biofuel stoves [24], and low building ventilation rates [25]. If prevention and remediation of dampness and mold in houses and other buildings were documented to substantially reduce some or all types of human respiratory infections, this would be good and important news.

The attributable risk proportion (ARP) of respiratory infections in the population associated with dampness or mold exposure would be estimated, assuming no confounding and that RRs approximate ORs, with formula (2):

(2) ARP=[Pe(RR−1)]/[Pe(RR−1)+1] 

[26] where: *Pe *is the proportion of the population exposed.

Based on a proportion of damp/moldy housing in the population of 20-50% [21], and selected ORs in Table 3, approximate ARPs would be: for acute bronchitis, 8-18%; for respiratory infections excluding common cold and nonspecific upper respiratory infections, 9-20%; and for respiratory infections in children or infants, 9-19%. Thus, if exposures related to residential dampness or mold directly caused respiratory infections, then preventing or remediating all this dampness and mold would reduce the prevalence of various respiratory infections by approximately 8-20%.

Thus, this review provides evidence that preventing or remediating dampness and mold in residences, a very common condition, may substantially reduce the burden of respiratory infections. This could be one of the few available preventive environmental strategies for these common diseases, now considered mostly inevitable. In addition, most exacerbations of asthma have been shown to occur in the presence of viral respiratory infections [27], and hospitalizations for severe exacerbations of asthma are strongly associated with viral infections [28]. This agrees with the finding that dampness and mold in buildings are associated consistently with asthma exacerbation [2,3]. Thus, reduction in viral respiratory infections may have important dual benefits.

## Conclusions

Dampness and mold in buildings are associated with moderate but statistically significant increases in respiratory infections and bronchitis. If these associations were causal, reducing dampness and mold in buildings would reduce the occurrence of respiratory infections, the most common human infections. The results of these meta-analyses provide support for recommendations by the Institute of Medicine and WHO to prevent building dampness and mold problems in buildings, and to take corrective actions where such problems occur. Additional focused research is necessary to document whether these associations are causal, and to develop more objective assessment tools for dampness, mold, or various other microbiologic factors that correlate with human health effects.

## List of abbreviations

Abbreviations used in this paper are: ARP: attributable risk proportion; CI: confidence interval; IOM: Institute of Medicine; OR: odds ratio; PE: proportion of the population exposed; RR: relative risk; SES: socioeconomic status; WHO: World Health Organization.

## Competing interests

The authors declare that they have no competing interests.

## Authors' contributions

WJF conceived the project, performed the literature review, abstracted the findings, and drafted the manuscript. EAE performed the statistical analyses, created the figures, and provided input on statistical questions. MJM assisted in the literature review and the analysis and interpretation of the data, and substantially revised the manuscript. All authors read and approved the final manuscript.

## Supplementary Material

Additional file 1 **Appendix 1 - Details of search strategy**. description of literature search, including specific search terms. Click here for file

## References

[B1] Bornehag CG Sundell J Bonini S Custovic A Malmberg P Skerfving S Sigsgaard T Verhoeff A Dampness in buildings as a risk factor for health effects, EUROEXPO: a multidisciplinary review of the literature (1998-2000) on dampness and mite exposure in buildings and health effects Indoor Air 2004 14 243 257 10.1111/j.1600-0668.2004.00240.x 15217478

[B2] Institute of Medicine Damp Indoor Spaces and Health 2004 Washington, D.C.: National Academies Press 25009878

[B3] World Health Organization Regional Office for Europe WHO Guidelines for Indoor Air Quality: Dampness and Mould WHO Guidelines for Indoor Air Quality Bonn, Germany 2009 http://www.euro.who.int/__data/assets/pdf_file/0017/43325/E92645.pdf 23785740

[B4] Fisk WJ Lei-Gomez Q Mendell MJ Meta-analyses of the associations of respiratory health effects with dampness and mold in homes Indoor Air 2007 17 284 296 10.1111/j.1600-0668.2007.00475.x 17661925

[B5] Antova T Pattenden S Brunekreef B Heinrich J Rudnai P Forastiere F Luttmann-Gibson H Grize L Katsnelson B Moshammer H Exposure to indoor mould and children's respiratory health in the PATY study Journal of Epidemiology and Community Health 2008 62 708 10.1136/jech.2007.065896 18621956

[B6] DerSimonian R Laird N Meta-analysis in clinical trials Control Clin Trials 1986 7 177 188 10.1016/0197-2456(86)90046-2 3802833

[B7] Stark PC Burge HA Ryan LM Milton DK Gold DR Fungal levels in the home and lower respiratory tract illnesses in the first year of life Am J Respir Crit Care Med 2003 168 232 237 10.1164/rccm.200207-730OC 12724122

[B8] Haverinen U Husman T Vahteristo M Koskinen O Moschandreas D Nevalainen A Pekkanen J Comparison of two-level and three-level classifications of moisture-damaged dwellings in relation to health effects Indoor Air 2001 11 192 199 10.1034/j.1600-0668.2001.011003192.x 11521504

[B9] van Gageldonk-Lafeber AB van der Sande MA Heijnen ML Peeters MF Bartelds AI Wilbrink B Risk factors for acute respiratory tract infections in general practitioner patients in The Netherlands: a case-control study BMC Infect Dis 2007 7 35 10.1186/1471-2334-7-35 17466060 PMC1871593

[B10] Biagini JM LeMasters GK Ryan PH Levin L Reponen T Bernstein DI Villareal M Khurana Hershey GK Burkle J Lockey J Environmental risk factors of rhinitis in early infancy Pediatr Allergy Immunol 2006 17 278 284 10.1111/j.1399-3038.2006.00386.x 16771781 PMC2233943

[B11] Bornehag CG Blomquist G Gyntelberg F Jarvholm B Malmberg P Nordvall L Nielsen A Pershagen G Sundell J Dampness in buildings and health. Nordic interdisciplinary review of the scientific evidence on associations between exposure to "dampness" in buildings and health effects (NORDDAMP) Indoor Air 2001 11 72 86 10.1034/j.1600-0668.2001.110202.x 11394014

[B12] Cooper RJ Hoffman JR Bartlett JG Besser RE Gonzales R Hickner JM Sande MA Principles of appropriate antibiotic use for acute pharyngitis in adults: background Ann Intern Med 2001 134 509 517 11255530 10.7326/0003-4819-134-6-200103200-00019

[B13] Vergison A Dagan R Arguedas A Bonhoeffer J Cohen R Dhooge I Hoberman A Liese J Marchisio P Palmu AA Otitis media and its consequences: beyond the earache The Lancet Infectious Diseases 2010 10 195 10.1016/S1473-3099(10)70012-8 20185098

[B14] Broor S Pandey RM Ghosh M Maitreyi RS Lodha R Singhal T Kabra SK Risk factors for severe acute lower respiratory tract infection in under-five children Indian Pediatr 2001 38 1361 1369 11752733

[B15] File TM Jr Marrie TJ Burden of community-acquired pneumonia in North American adults Postgrad Med 2010 130 141 10.3810/pgm.2010.03.2130 20203464

[B16] McChlery S Ramage G Bagg J Respiratory tract infections and pneumonia Periodontol 2000 2009 49 151 165 10.1111/j.1600-0757.2008.00278.x 19152532 PMC7168030

[B17] Penttinen P Huttunen K Pelkonen J Hirvonen MR The proportions of Streptomyces californicus and Stachybotrys chartarum in simultaneous exposure affect inflammatory responses in mouse RAW264.7 macrophages Inhalation Toxicology 2005 17 79 85 10.1080/08958370590903004 15764485

[B18] Yli-Pirilä T Huttunen K Nevalainen A Seuri M Hirvonen MR Effects of co-culture of amoebae with indoor microbes on their cytotoxic and proinflammatory potential Environmental Toxicology 2007 22 357 367 10.1002/tox.20274 17607727

[B19] McGwin G Lienert J Kennedy JI Formaldehyde exposure and asthma in children: a systematic review Environmental Health Perspect 2010 118 313 317 10.1289/ehp.0901143 PMC2854756 20064771

[B20] Matthews TG Fung KW Tromberg BJ Hawthorne AR Impact of indoor environmental parameters on formaldehyde concentrations in unoccupied research houses Journal of the Air Pollution Control Association 1986 36 1244 1249 3794086 10.1080/00022470.1986.10466172

[B21] Mudarri D Fisk WJ Public health and economic impact of dampness and mold Indoor Air 2007 17 226 235 10.1111/j.1600-0668.2007.00474.x 17542835

[B22] Birch EE Khoury JC Berseth CL Castaneda YS Couch JM Bean J Tamer R Harris CL Mitmesser SH Scalabrin DM The impact of early nutrition on incidence of allergic manifestations and common respiratory illnesses in children J Pediatr 2010 156 902 906 906 e901 10.1016/j.jpeds.2010.01.002 20227721

[B23] Moritsugu KP The 2006 Report of the Surgeon General: the health consequences of involuntary exposure to tobacco smoke American Journal of Preventive Medicine 2007 32 542 10.1016/j.amepre.2007.02.026 17533072

[B24] Morris K Morganlander M Coulehan JL Gahagen S Arena VC Wood-burning stoves and lower respiratory tract infection in American Indian children Archives of Pediatrics and Adolescent Medicine 1990 144 105 10.1001/archpedi.1990.02150250117047 2294707

[B25] Milton DK Glencross PM Walters MD Risk of sick leave associated with outdoor air supply rate, humidification, and occupant complaints Indoor Air 2000 10 212 221 10.1034/j.1600-0668.2000.010004212.x 11089326

[B26] Rockhill B Newman B Weinberg C Use and misuse of population attributable fractions American Journal of Public Health 1998 88 15 10.2105/AJPH.88.1.15 9584027 PMC1508384

[B27] Rosenthal LA Avila PC Heymann PW Martin RJ Miller EK Papadopoulos NG Peebles RS Gern JE Viral respiratory tract infections and asthma: the course ahead J Allergy Clin Immunol 2010 125 1212 1217 10.1016/j.jaci.2010.04.002 20513518 PMC2880817

[B28] Johnston SL Pattemore PK Sanderson G Smith S Campbell MJ Josephs LK Cunningham A Robinson BS Myint SH Ward ME Tyrrell DA Holgate ST The relationship between upper respiratory infections and hospital admissions for asthma: a time-trend analysis Am J Respir Crit Care Med 1996 154 654 660 8810601 10.1164/ajrccm.154.3.8810601

[B29] Bakke JV Norback D Wieslander G Hollund BE Moen BE Pet keeping and dampness in the dwelling: associations with airway infections, symptoms, and physiological signs from the ocular and nasal mucosa Indoor Air 2007 17 60 69 10.1111/j.1600-0668.2006.00455.x 17257153

[B30] Brunekreef B Dockery DW Speizer FE Ware JH Spengler JD Ferris BG Home dampness and respiratory morbidity in children Am Rev Respir Dis 1989 140 1363 1367 2817598 10.1164/ajrccm/140.5.1363

[B31] Dales RE Zwanenburg H Burnett R Franklin CA Respiratory health effects of home dampness and molds among Canadian children Am J Epidemiol 1991 134 196 203 1862803 10.1093/oxfordjournals.aje.a116072

[B32] Diez U Rehwagen M Rolle-Kampczyk U Wetzig H Schulz R Richter M Lehmann I Borte M Herbarth O Redecoration of apartments promotes obstructive bronchitis in atopy risk infants--results of the LARS Study Int J Hyg Environ Health 2003 206 173 179 10.1078/1438-4639-00218 12872525 PMC7129632

[B33] du Prel X Kramer U Behrendt H Ring J Oppermann H Schikowski T Ranft U Preschool children's health and its association with parental education and individual living conditions in East and West Germany BMC Public Health 2006 6 312 10.1186/1471-2458-6-312 17194300 PMC1769487

[B34] Ekici M Ekici A Akin A Altinkaya V Bulcun E Chronic airway diseases in adult life and childhood infections Respiration 2007 75 55 59 10.1159/000102952 17505127

[B35] Karevold G Kvestad E Nafstad P Kvaerner KJ Respiratory infections in schoolchildren: co-morbidity and risk factors Arch Dis Child 2006 91 391 395 10.1136/adc.2005.083881 16464964 PMC2082748

[B36] Kilpelainen M Terho EO Helenius H Koskenvuo M Home dampness, current allergic diseases, and respiratory infections among young adults Thorax 2001 56 462 467 10.1136/thorax.56.6.462 11359962 PMC1746066

[B37] Koskinen OM Husman TM Meklin TM Nevalainen AI Adverse health effects in children associated with moisture and mold observations in houses International Journal of Environmental Health Research 1999 9 143 156 10.1080/09603129973281

[B38] Li CS Hsu LY Home dampness and childhood respiratory symptoms in a subtropical climate Arch Environ Health 1996 51 42 46 10.1080/00039896.1996.9935992 8629862

[B39] Pettigrew MM Gent JF Triche EW Belanger KD Bracken MB Leaderer BP Association of early-onset otitis media in infants and exposure to household mould Paediatr Perinat Epidemiol 2004 18 441 447 10.1111/j.1365-3016.2004.00596.x 15535820

[B40] Pirhonen I Nevalainen A Husman T Pekkanen J Home dampness, moulds and their influence on respiratory infections and symptoms in adults in Finland Eur Respir J 1996 9 2618 2622 10.1183/09031936.96.09122618 8980978

[B41] Rylander R Megevand Y Environmental risk factors for respiratory infections Arch Environ Health 2000 55 300 303 10.1080/00039890009604021 11063404

[B42] Spengler J Neas L Nakai S Dockery D Speizer F Ware J Raizenne M Respiratory Symptoms and Housing Characteristics Indoor Air 1994 4 72 82 10.1111/j.1600-0668.1994.t01-2-00002.x

[B43] Spengler JD Jaakkola JJ Parise H Katsnelson BA Privalova LI Kosheleva AA Housing characteristics and children's respiratory health in the Russian Federation Am J Public Health 2004 94 657 662 10.2105/AJPH.94.4.657 15054021 PMC1448314

[B44] Strachan DP Damp housing and childhood asthma: validation of reporting of symptoms BMJ 1988 297 1223 1226 10.1136/bmj.297.6658.1223 3145060 PMC1834724

[B45] Sun Y Zhang Y Sundell J Fan Z Bao L Dampness in dorm rooms and its associations with allergy and airways infections among college students in China: a cross-sectional study Indoor Air 2009 19 348 356 10.1111/j.1600-0668.2009.00614.x 19627367

[B46] Yang CY Chiu JF Chiu HF Kao WY Damp housing conditions and respiratory symptoms in primary school children Pediatr Pulmonol 1997 24 73 77 10.1002/(SICI)1099-0496(199708)24:2<73::AID-PPUL1>3.0.CO;2-J 9292897

[B47] Yang CY Effects of indoor environmental factors on risk for acute otitis media in a subtropical area Journal of Toxicology and Environmental Health, Part A 1999 56 111 119 10.1080/009841099158178 9972922

