# Supplementary material for: Association of residential dampness and mold with respiratory tract infections and bronchitis: a meta-analysis
Source: Environ Health. 2010 Nov 15;9:72. doi: 10.1186/1476-069X-9-72 (PMC3000394; doi:10.1186/1476-069X-9-72)
Supplement: Additional file 1 — Appendix 1 - Details of search strategy. description of literature search, including specific search terms. [file 1476-069X-9-72-S1.DOC]

**Appendix 1 - Details of search strategy**

Search 1 – online search of PubMed

http://www.ncbi.nlm.nih.gov/sites/entrez?tool=cdl&holding=lbllib

(1,092 citations retrieved – 18 eligible)

search string:

(dampness OR damp OR “water damage” OR mold OR mildew OR leaks OR condensation OR moisture OR water OR fungi OR fungal OR mould OR microbial)

AND (respiratory infection OR “lung infection” OR bronchial OR bronchitis OR "common cold" OR "otitis media" OR "ear infection" OR sinusitis OR "sinus infection" OR tonsillitis OR tonsillopharyngitis OR croup OR bronchiolitis OR "chest cold" OR pneumonia)

AND (house OR indoor OR home OR residence OR apartment OR dwelling OR "living unit" OR residential)

NOT (aspergillosis OR pneumocystis OR "Mycobacterium avium" OR legionnaire OR legionnaires OR legionella OR Pontiac);

Limits: English, human only.

Search 2 – online search of the journal Indoor Air (http://www3.interscience.wiley.com/journal/118513116/home)

(40 citations retrieved, 1 additional eligible – Spengler 1994))

search string:

(dampness or moisture or mold or mildew) AND (home or house or housing) and (respiratory or health)

Search 3 - manual search of reference list in WHO Guidelines on Dampness, Mold, and Health [3] – 4 additional citations selected (Brunekreef 1989, du Prel 2006, Koskinen 1999, Li 1996).
